# Would the Brazilian population support the alcohol policies recommended by the World Health Organization?

**DOI:** 10.11606/s1518-8787.2022056004093

**Published:** 2022-06-28

**Authors:** Raquel B. De Boni, Jurema C. Mota, Carolina Coutinho, Francisco I. Bastos

**Affiliations:** I Fundação Oswaldo Cruz Instituto de Comunicação e Informação Cientifica e Tecnológica em Saúde Rio de Janeiro RJ Brasil Fundação Oswaldo Cruz. Instituto de Comunicação e Informação Cientifica e Tecnológica em Saúde. Rio de Janeiro, RJ, Brasil; II Fundação Getúlio Vargas Escola de Administração de Empresas São Paulo SP Brasil Fundação Getúlio Vargas. Escola de Administração de Empresas. São Paulo, SP, Brasil

**Keywords:** Alcohol Drinking, prevention & control, Products Publicity Control, Public Policy, Public Opinion, Brazil

## Abstract

**OBJECTIVE:**

To evaluate the support of the Brazilian population to the alcohol-policies proposed by the World Health Organization to decrease alcohol harm (specifically: to decrease alcohol availability and advertising, and to increase pricing). In addition, we evaluated the factors associated with being against those policies.

**METHODS:**

Data from 16,273 Brazilians, aged 12–65 years, interviewed in the 3^rd^ Brazilian Household Survey on Substance Use (BHSU-3) were analyzed. The BHSU-3 is a nationwide, probability survey conducted in 2015. Individuals were asked if they would be against, neutral, or in favor of seven alcohol policies grouped as: 1) Strengthen restrictions on alcohol availability; 2) Enforce bans or restrictions on alcohol advertising, sponsorship, and promotion; and 3) Raise prices on alcohol through excise taxes and pricing. Generalized linear models were fitted to evaluate factors associated with being against each one of those policies and against all of policies.

**RESULTS:**

Overall, 28% of the Brazilians supported all the above mentioned policies, whereas 16% were against them. The highest rate of approval refers to restricting advertising (53%), the lowest refers to increasing prices (40%). Factors associated with being against all policies were: being male (AOR = 1.1; 95%CI: 1.0–1.3), not having a religion (AOR = 1.4; 95%CI: 1.1–1.8), being catholic (AOR = 1.3; 95%CI: 1.1–1.5), and alcohol dependence (AOR = 1.6; 95%CI: 1.1–2.4).

**CONCLUSIONS:**

The Brazilian government could count on the support of most of the population to restrict alcohol advertising. This information is essential to tackle the lobby of the alcohol industry and its clever marketing strategy.

## INTRODUCTION

Decreasing the harmful use of alcohol is a specific objective of the Sustainable Development Goals (SDGs) since it is a leading risk factor associated with an increased burden of disease worldwide, and the leading risk factor for mortality among people aged 15–49 years^1^ . In Brazil, the prevalence of alcohol use in the last 12 months has been estimated at 43.1%, while the prevalence of binge drinking and alcohol dependence were estimated at 16.5% and 1.5%, respectively^4^ . Accordingly, alcohol use is estimated to be the 5^th^ most relevant risk factor for death and disability in the country^5^ .

Alcohol policies are effective to decrease alcohol-related problems^6^ . In 2010, the World Health Organization (WHO)’s Global Strategy to Reduce the Harmful Use of Alcohol^7^ recommended policies and interventions in ten target areas “(a) leadership, awareness and commitment, b) health services’ response, c) community action, d) drink-driving policies and countermeasures, e) availability of alcohol, f) marketing of alcoholic beverages, g) pricing policies, h) reducing the negative consequences of drinking and alcohol intoxication, i) reducing the public health impact of illicit alcohol and informally produced alcohol, and j) monitoring and surveillance.” In 2018, the WHO launched the SAFER initiative to help governments implement five of the most cost-effective policies^8^ . SAFER is the acronym for “Strengthen restrictions on alcohol availability”, “Advance and enforce drink driving counter measures”, “Facilitate access to screening, brief interventions and treatment”, “Enforce bans or comprehensive restrictions on alcohol advertising, sponsorship, and promotion”, and “Raise prices on alcohol through excise taxes and pricing policies”^9^ . Three of those policies are also recommended as “best buys” to tackle non-communicable diseases (increase prices, restrict advertising, and restrict physical availability)^10^ .

Such policies are sound and feasible, but implementation demand the government’s commitment with public health and consistency in confronting the powerful industry lobby. Partnership with the civil society should be assessed and explored in detail. The putative coalition of government and the civil society is key to remove vetoes imposed by different lobbies, especially the one fostered by the alcohol industry^11^ .

Alcohol policies have evolved in Brazil in the last 20 years, although many gaps persist^12 , 13^ . For instance, in 2010, a new legislation on drinking and driving was approved, in which the legal amount of alcohol blood concentration allowed for driving was decreased to zero and the random breath tests to be performed were increased^14^ . Restrictions on alcohol sales were implemented for specific events and on petrol stations. There are no alcohol monopolies, and although a license is required to sell alcohol, almost every commercial establishment is able to get one. Additionally, alcohol is largely sold in the informal market and there is no regulation on in/out premise drinking or alcohol density outlets^15 , 16^ . Regarding advertising policies, there are no regulations on alcohol industry sponsorship, alcohol sales promotion, and beer advertising. Finally, regarding pricing, there are excise taxes for beer, wine, and spirits^1^ – but they are modest compared to other countries and are not clear to the general population.

It is reasonable to believe that governments from democratic countries would be more prone to implement policies supported by the general population. International studies have found that targeted policies – such as legal age to purchase alcoholic beverages and drinking and driving control – are more likely to be supported than general policies (such as increasing tax and prices)^17 , 18^ . Moreover, support levels varied by age, gender, income level, and pattern of alcohol use^17^ .

We aim to evaluate the support of the Brazilian population regarding policies targeted toward decreasing alcohol availability and advertising, as well as toward increasing prices. Such policies have shown to be effective and cost-effective in different settings^19^ . Additionally, we evaluated the factors associated with being against those policies.

## METHODS

This is a secondary analysis of data collected in the 3^rd^Brazilian Household Survey on Substance Use (BHSU-3). The full methodological report describing the sampling design, questionnaires, data collection, data entry, handling of non-response and estimation procedures is publicly available at https://www.arca.fiocruz.br/handle/icict/34614 (in Portuguese, English, and Spanish). Briefly, the BHSU-3 was a nationwide, probability sample survey conducted in 2015. The study population comprised 16,273 Brazilians, 12–65 years old. Native individuals living in indigenous villages, inmates, and individuals with physical or mental disabilities that may preclude answering the interviews were not eligible. Individuals were interviewed face-to-face by trained interviewers after signing the informed consent/assent form. The study was approved by the institutional review board of the *Escola Politécnica Joaquim Venâncio* -Fiocruz (CAAE # 35283814.4.0000.5241)^4 , 20^ .

### Outcomes

Individuals were asked if they would be in favor, neutral, or against, in case the following policies were implemented to decrease alcohol-related harm: reducing alcohol outlets, restricting opening hours, implementing licenses to sell alcohol, controlling advertising, forbidding sponsoring sports events, increasing price, and increasing taxes. Seven dichotomic outcomes were created for the present analysis. Initially we defined the three major policies to be evaluated (following the WHO’s “best buys” included in the SAFER initiative):

1. Strengthen restrictions on alcohol availability: included the questions on *Reduce alcohol outlets policy, Restrict opening hours policy* , and *Implement license to sell alcohol policy* ;
2. Enforce bans or restrictions on alcohol advertising, sponsorship, and promotion: included *Control advertising policy* and *Forbid sponsoring sports policy* ;
3. Raise prices on alcohol through excise taxes and pricing: included *Increase taxes policy* and *Increase price policy* .

To evaluate the frequencies of being “In favor” of each one of the above, individuals had to answer they were in favor to all the policies related to the group. Those who were neutral or against them were defined, for the sake of our study, as “not in favor.” Likewise, to be considered “against” each policy, individuals had to answer they were against all the policies from the group.

Finally, the main outcome “Being against all policies” was created as follows: *Strengthen restrictions on alcohol availability policies* was equal to “against” AND *Enforce bans or restrictions on alcohol advertising, sponsorship, and promotion policies* was equal to “against” AND *Raise prices on alcohol through excise taxes and pricing policies* was equal to “against.” Thus, “Being against all policies” was “yes,” and everything else was “no”.

### Independent Variables

**Demographic variables:** were collected using questions from the Brazilian Demographic Census^25^ , and included: sex at birth (male *versus* female), color/race (white, black, mixed, other), educational level (primary, high-school, or college/university), income, religion (Catholic, Christian, none, other), reporting a steady partner (yes, no), number of children (none, one or more), Brazil’s geographic macroregions (North/ Northeast, Southeast/ South, and Center-west), living in urban *versus* rural areas.

**Self-rated health** : was evaluated by the question “Overall, how is your health?” Possible answers were provided using a Likert scale ranging from “very bad” to “very good”, which were categorized as very good/good, regular/don’t know, and very bad/bad^26^ .

**Alcohol use in the last 12 months:** it is reported as composite variable “no use in the last 12 months”, “use but no dependence”, and “alcohol dependence”. Alcohol dependence was evaluated using the Diagnostic and Statistical Manual of Mental Disorders criteria^4 , 27^ .

**Substance use in the last 12 months:** cannabis and cocaine were included since they are the most frequent illicit substances used in the country^28^ .

### Statistical Analysis

Point prevalence and respective standard errors of being in favor of each group of policies (i.e. “Strengthen restrictions on alcohol availability policies”, “Enforce bans or restrictions on alcohol advertising, sponsorship, and promotion”, and “Raise prices on alcohol through excise taxes and pricing policies”) were calculated for the whole sample, as well, as stratified by the independent variables.

Generalized linear regression models were fitted to evaluate factors independently associated with “being against all policies” and being against each subset of policies. Initially, bivariate analysis was conducted to evaluate the association of independent variables and each one of the outcomes. Variables presenting association at p-value < 0.20 at the bivariate analysis were considered for the multivariable regression models. Four backward logistic models were fitted, until reaching the most parsimonious models. All analyses considered sample weights, design effect, and weight calibration. Analyses were carried out using R v.3.5.0 using the ‘survey’ and ‘surveyer’ libraries^29^ .

## RESULTS

The 16,273 individuals interviewed represent an estimated population of 153 million Brazilians. Overall, 27.6% (SE 1.0) were in favor of all alcohol-related policies. 53% of the population were in favor of enforcing bans or restrictions on alcohol advertising, while 40% were in favor of raising prices. The higher rates of support for all policies were reported by females, Christian, those with a steady partner, with children, living in the North/Northeast, reported bad/very bad self-rated health, and who did not use alcohol, cannabis, or cocaine ( Table 1 ).

**Table 1:** Proportion (in %) of individuals in favor of alcohol policies by demographic characteristics, self-rated health, and substance use. Brazil, 2015

SAFER	Total	Strengthen restrictions on alcohol availability	Enforce bans or restrictions on alcohol advertising, sponsorship, and promotion	Raise prices on alcohol through excise taxes and pricing policies
Sample size (n)	16,723	6,593	8,762	6,531
Estimated population (N) x 1,000	153,095,166	62,070,445 (40.5)	80,681,192 (52.7)	61,161,300 (40.0)
Characteristic	Estimated population (%)	Prevalence (SE)	Prevalence (SE)	Prevalence (SE)
Sex				
Male	74,179,205 (48.4)	36.0 (1.3)	49.8 (1.3)	36.7 (1.2)
Female	78,915,961 (51.6)	44.9 (1.3)	55.4 (1.2)	43.1 (1.2)
Age				
12–17 years	20,276,385 (13.2)	45.9 (2.6)	47.7 (2.8)	41.2 (2.5)
18–44 years	84,373,066 (55.1)	39.5 (1.1)	52.1 (1.1)	38.5 (1.1)
≥ 45 years	48,445,715 (31.6)	40.1 (1.3)	55.8 (1.3)	42.0 (1.3)
Educational level				
Primary	90,065,490 (58.8)	43.5 (1.4)	51.6 (1.4)	41.6 (1.3)
High school	47,631,405 (31.1)	37.7 (1.2)	53.5 (1.2)	37.8 (1.2)
College or more	15,398,271 (10.1)	32.1 (2.0)	56.6 (1.7)	37.0 (2.3)
Color/race				
White	67,777,519 (44.3)	37.9 (1.2)	52.9 (1.2)	38.3 (1.2)
Black	15,497,481 (10.1)	41.3 (2.0)	51.0 (2.2)	40.2 (2.2)
Mixed (Biracial)	68,083,270 (44.5)	42.9 (1.6)	52.9 (1.6)	41.5 (1.5)
Other	1,736,896 (11.3)	43.8 (5.3)	52.2 (5.1)	39.3 (5.3)
Monthly family income				
> R$ 1,500.00	80,231,542 (52.4)	42.9 (1.6)	50.8 (1.5)	41.2 (1.4)
≥ R$ 1,501.00	72,863,625 (47.6)	38.0 (1.1)	54.8 (1.1)	38.6 (1.2)
Religion				
None	13,174,180 (8.6)	29.8 (2.0)	47.6 (2.1)	29.9 (1.8)
Catholic	91,242,525 (60.0)	38.4 (1.3)	50.5 (1.3)	38.9 (1.2)
Christian	42,892,303 (28.0)	49.0 (1.4)	58.4 (1.5)	45.7 (1.5)
Other	5,786,158 (4.0)	36.7 (2.6)	57.0 (2.5)	37.2 (2.9)
Reporting a steady partner				
Yes	67,571,165 (55.9)	41.1 (1.4)	55.8 (1.3)	41.5 (1.4)
No	85,524,001 (44.1)	40.1 (1.2)	50.3 (1.2)	38.7 (1.1)
Number of children				
None	55,128,003 (36.0)	38.3 (1.3)	49.1 (1.5)	36.9 (1.3)
≥ 1	97,967,163 (64.0)	41.8 (1.2)	54.8 (1.2)	41.7 (1.2)
Geographic macroregion				
North/Northeast	54,348,090 (35.5)	46.0 (2.1)	53.0 (2.0)	44.7 (1.9)
South/Southeast	87,127,839 (56.9)	37.0 (1.4)	52.6 (1.4)	37.7 (1.5)
Center-West	11,619,236 (7.6)	41.4 (2.9)	52.3 (3.1)	34.7 (2.1)
Living in urban or rural areas				
Urban	126,691,582 (82.7)	39.8 (1.0)	53.1 (1.0)	39.0 (1.1)
Rural	26,403,584 (17.3)	44.3 (2.9)	50.8 (2.8)	44.7 (2.6)
Self-rated Health				
Very good/good	111,852,986 (73.1)	39.2 (1.1)	52.1 (1.1)	38.8 (1.1)
Regular/don´t know	35,072,470 (22.9)	43.8 (1.6)	54.1 (1.6)	42.7 (1.5)
Very bad/bad	6,169,710 (4.0)	47.1 (3.5)	54.8 (3.2)	45.4 (3.5)
Alcohol use (12 months)				
No	87,151,956 (56.9)	47.1 (1.4)	56.4 (1.3)	45.6 (1.3)
Yes – no dependence	63,615,148 (41.6)	31.8 (1.1)	48.0 (1.2)	32.5 (1.1)
Alcohol dependence	2,328,062 (1.5)	34.4 (3.5)	42.9 (4.1)	29.8 (3.7)
Cannabis use (12 months)				
Yes	3,865,259 (2.5)	19.7 (2.8)	44.4 (3.9)	19.2 (2.7)
No	149,229,907 (97.5)	41.1 (1.1)	52.9 (1.1)	40.5 (1.1)
Cocaine use (12 months)				
Yes	1,339,656 (0.9)	27.4 (5.0)	50.1 (5.6)	30.7 (5.0)
No	151,755,510 (99.1)	40.7 (1.1)	52.7 (1.1)	40.0 (1.1)
In favor of all the policies (N - % (SE)		42,337,057 (27.6 ( 1.0))		

SE: standard error.

**Figure 1:**
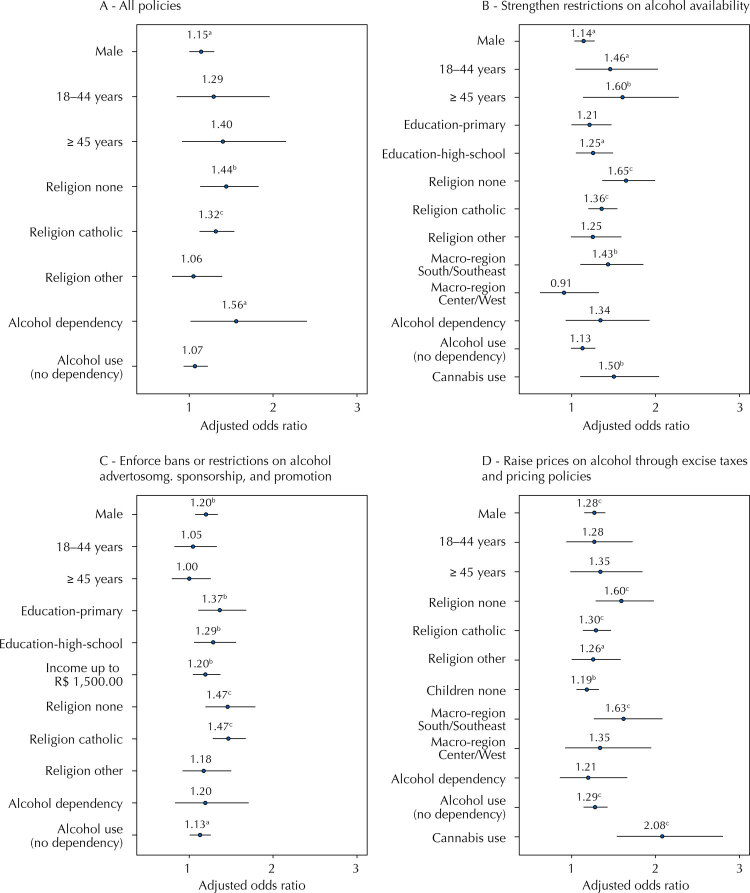
Generalized linear regression models to evaluate factors associated with being against a) All policies, b) Strengthening restriction on alcohol availability, c) Enforcing bans or restrictions on alcohol advertising, sponsorship, and promotion, d) Raising prices on alcohol through excise taxes and pricing policies. Brazil, 2015.

^a^ p-value < 0.05

^b^ p-value < 0.01

^c^ p-value < 0.001

Overall, 15.8% (0.9) of the Brazilians were against all alcohol policies: 25.3% were against the strengthening of restrictions on alcohol availability; 28.5% were against the enforcement of bans or restrictions on alcohol advertising, sponsorship, and promotion; and 30.6% were against the raising of prices on alcohol by excise taxes and pricing policies. Figure 1 and Table 2 show the factors associated with being against all policies and specifically against each subset of policies. The only factors associated with being against all policies and all subsets of policies were being male, not having a religion, or being Catholic (compared to Christian). Age above 44 years was associated with being against restrictions on alcohol availability. To live in the South/Southeast (most urbanized and industrialized) regions (compared to North/Northeast) was associated with being against restrictions on alcohol availability and price increase. Having primary education ( *versus* college education or more) increased the chance of being against advertising control. Alcohol dependence increased the likelihood of being against all policies, while alcohol use in the last 12 months increased the likelihood of being against advertising and price increase. Finally, cannabis use in the last 12 months was associated with being against restrictions on availability and pricing.

**Table 2:** Generalized linear regression models to evaluate factors associated with being against: a) All policies, b) Strengthening restriction on alcohol availability, c) Enforcing bans or restrictions on alcohol advertising, sponsorship, and promotion, d) Raising prices on alcohol through excise taxes and pricing policies. Brazil, 2015.

Characteristic	All policies	Strengthen restrictions on alcohol availability	Enforce bans or restrictions on alcohol advertising, sponsorship, and promotion	Raise prices on alcohol through excise taxes and pricing policies
	AOR (95%CI)	AOR (95%CI)	AOR (95%CI)	AOR (95%CI)
Male	1.15 (1.01–1.30)	1.14 (1.03–1.27)	1.20 (1.08–1.34)	1.28 (1.16–1.41)
18–44 years	1.29 (0.86–1.96)	1.46 (1.05–2.03)	1.05 (0.82–1.34)	1.28 (0.94–1.73)
≥ 45	1.40 (0.91–2.16)	1.60 (1.13–2.28)	1.00 (0.80–1.26)	1.35 (0.99–1.85)
Education Primary		1.21 (1.00–1.47)	1.37 (1.11–1.69)	
Education High school		1.25 (1.05–1.50)	1.29 (1.06–1.56)	
Income up to R$1,500.00			1.20 (1.05–1.38)	
Religion none	1.44 (1.14–1.83)	1.65 (1.36–1.99)	1.47 (1.20–1.79)	1.60 (1.29–1.99)
Religion Catholic	1.32 (1.13–1.54)	1.36 (1.20–1.54)	1.47 (1.28–1.68)	1.30 (1.14–1.48)
Religion other	1.06 (0.80–1.40)	1.25 (0.99–1.59)	1.18 (0.92–1.50)	1.26 (1.00–1.59)
Children none				1.19 (1.07–1.33)
Macroregion South/Southeast		1.43 (1.10–1.85)		1.63 (1.27–2.09)
Macroregion Center-West		0.91 (0.62–1.32)		1.35 (0.93–1.96)
Alcohol dependency	1.56 (1.01–2.41)	1.34 (0.93–1.93)	1.20 (0.84–1.72)	1.21 (0.87–1.67)
Alcohol use (no dependency)	1.07 (0.94–1.23)	1.13 (0.99–1.28)	1.13 (1.01–1.26)	1.29 (1.15–1.44)
Cannabis use		1.50 (1.10–2.04)		2.08 (1.55–2.81)

AOR: adjusted odds ratio; 95%CI: 95% confidence interval.

**Figure 2:**
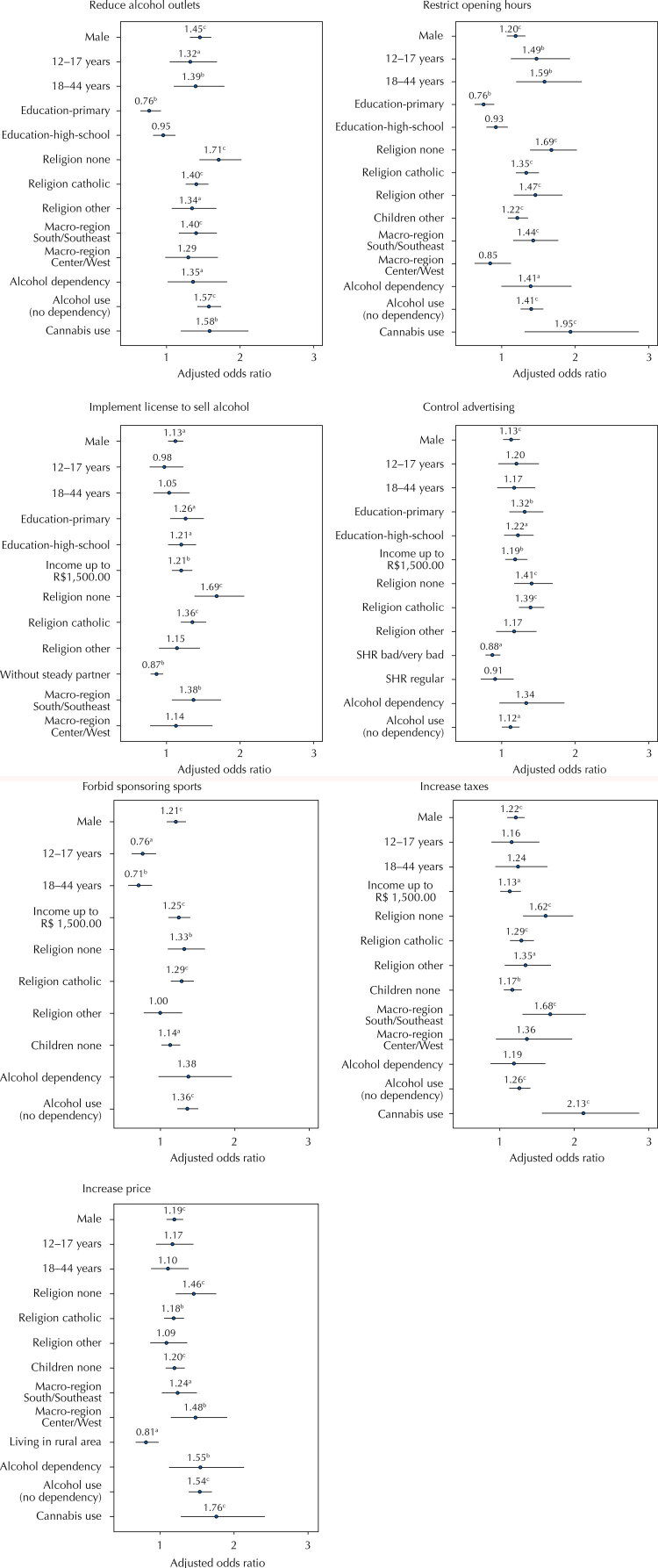
Generalized linear regression models to evaluate factors associated with being against each alcohol related policy in Brazil, 2015.

^a^ p-value < 0.05

^b^ p-value < 0.01

^c^ p-value < 0.001

Interestingly, after controlling for confounding factors, having children and having used cocaine in the last 12 months were not found to be associated to any of the outcomes. Figure 2 shows the factors associated with each one of the abovementioned policies.

## DISCUSSION

The present study shows that 28% of the Brazilian population was in favor of implementing all alcohol-related policies evaluated. Policies related to reducing advertisement were endorsed by most of the population. On the other hand, 16% of the population was against implementing all policies. Factors associated with being against implementation were being male, not having a religion or being catholic, and presenting alcohol dependence.

Overall, the support for alcohol public policies in Brazil (40%–52%) was similar to the United States (33%–72%) and Canada (35%–68%), but lower than other middle-high income countries, such as India (80%–86%) and China (57%–85%)^17^ . This level of putative endorsement should be further explored via triangulation with further assessments using in-depth interviews and focus groups, among other methods^30^ .

Notably, alcohol-related policies are less restrictive and loosely enforced in Brazil compared to the United States/Canada. Currently, there are no regulations on alcohol outlets availability, policies regarding prices are not transparent, and beer advertising is permitted. Such permissiveness is not compatible with the high rates of alcohol-related mortality in the country. In addition to the fact that alcohol drinking is the 5^th^ risk factor regarding the overall burden of disease^5^ , Brazil ranks second, in the Americas (after the United States), on the absolute number of deaths that were 100% attributable to alcohol use^31^ .

Changing this situation, unfortunately, seems to be a distant dream. Brazil is still considered a market in expansion by the alcohol industry, which, in 2011, was one of the main investors in marketing in the country. Such industry, as well as the tobacco and the ultraprocessed food industries (known as “unhealthy commodity industry”^32^ ), has a wide range of common strategies to influence public policies and maintain their profits^33^ . Those include both “coercion and appeasement” strategies^36^ . In 2018, a systematic review showed that the two main strategies used by the alcohol industry were of excluding from the debate issues against their interest (like increasing prices) and building relationships with different agents who could influence policy making (including researchers)^37^ .

The characteristics of individuals who support the policies are similar to those from other countries^18 , 38 , 39^ , including sex, religion (Christian), and pattern of alcohol use (abstainers). Other characteristics, such as being a parent and not using cannabis or cocaine, are less studied elsewhere. Individuals raising children may be more likely to be concerned about their futures, especially because traffic crashes and violence (which are both related to alcohol consumption) are the main causes of death among male young adults in the country^5^ .

Furthermore, marketing strategies are aggressive toward adolescents^40^ − who are particularly vulnerable to propaganda^41^ − which may increase parents’ concerns. Restricting alcohol advertising is an effective measure to prevent alcohol harms. For instance, since 1975 alcohol advertising was banned in Norway, and alcohol sales have decreased over time^42^ . Identifying groups who would putatively support these policies may be important to find community allies seeking to reduce alcohol consumption and harms^38^ .

The characteristics of individuals who were against alcohol policies (male, alcohol use, and alcohol dependence) were also similar to other studies^17^ . Notably, men are less likely to support alcohol policies, despite alcohol-related mortality among males being higher than among their female counterparts^31^ . One possible explanation is their historical higher use of alcohol (compared to women). In this sense, they could consider alcohol drinking as less harmful. Alcohol policies, in fact, tend to strongly affect men, as recent studies from Lithuania and Poland have shown^43 , 44^ . Similarly, it is possible that alcohol users/people who are alcohol-dependent have a lower risk perception regarding alcohol drinking. Considering that these individuals could be benefited by policies regarding screening and treatment, they may be more prone to see such policies as needed^38^ . Since we did not collect this information in our survey, further research is necessary in Brazil.

The policy group with lower rate of support was the one related to raising prices and taxes, as many other studies have also shown^17 , 38 , 39^ . To raise price is one of the most effective public health policies for decreasing consumption of harmful products. Two systematic reviews evaluated empirical studies regarding the effect of increasing taxes on goods that represent risk to the public health, i.e.: tobacco, alcohol, sugar-sweetened beverages showing the benefit of those policies^45 , 46^ . Interestingly very few studies related to alcohol use were included and the reasons for this low number were not discussed. Additionally, WHO clearly recommends tax rates for tobacco (70%) and sugar-sweetened beverages (20%), but not for alcohol. As recently stated, alcohol remains the “blind spot in global health”^47^ . The reasons for that include the powerful lobby of alcohol industry across governments and countries, as well as its sophisticated marketing strategy^47^ . Restricting or banning alcohol advertising is one of the most cost-effective policies to decrease alcohol drinking and its positive results on decreasing sales have been documented for a long time^42^ . However, controlling advertising is getting even harder due to social media. Findings from a study on browsing contents disseminated by Twitter® show that the most frequent arguments used by alcohol industry/supporters relate to the idea that liberal policies would increase revenue, individual liberties must be prioritized over government control, and education is the best solution for alcohol related problems^51^ .

It is worrisome that there are researchers and opinion makers who still think this way. Among the huge challenges ahead remains the need to create population demand, instead of general agreement, of effective public policies^48^ . Such task is possible, as can be seen by the demand created for HIV pre-exposure prophylaxis and HIV self-testing – which, in less than five years, became freely available in the Brazilian Public Health System.

This study is not free of limitations. It is impossible to exclude social desirability bias, which could have increased the prevalence of people in favor of alcohol policies. Causality may not be inferred from the observed associations due to the cross-sectional design. Brazil lacks historical series regarding population opinion on alcohol/substance use perceptions and policies which could be useful in case of their actual implementation. Moreover, questions regarding policies related to drinking and driving, and alcohol screening/treatment were not performed in the survey. Additional questions are thus necessary to evaluate public opinion on every aspect of the SAFER initiative.

In conclusion, Brazilian government would count with the support of most of the population to implement and enforce restrictions on alcohol advertising, sponsorship, and promotion. However, strong political will and transparency are essential to tackle the strong lobby of the alcohol industry within this endeavor. The permanent partnership between government, at the national & subnational levels, and the civil society is key. Representatives and policymakers are sensible to the pressures of the alcohol industry (some of them quite subtle and hard to discern)^52^ , but they should be, and usually are, also sensible to the points of view of their constituents and future voters.
